# Placebo-Controlled Trials in Surgery: A Systematic Review and Meta-Analysis

**DOI:** 10.1097/MD.0000000000003516

**Published:** 2016-04-29

**Authors:** Pascal Probst, Kathrin Grummich, Julian C. Harnoss, Felix J. Hüttner, Katrin Jensen, Silvia Braun, Meinhard Kieser, Alexis Ulrich, Markus W. Büchler, Markus K. Diener

**Affiliations:** From the Department of General, Visceral and Transplantation Surgery (PP, JCH, FJH, AU, MWB, MKD), University of Heidelberg; The Study Center of the German Surgical Society (SDGC); University of Heidelberg (PP, KG, JCH, SB, MKD); and Institute of Medical Biometry and Informatics (KJ, MK), University of Heidelberg, Heidelberg, Germany.

## Abstract

This systematic review was performed to investigate the ethical justification, methodological quality, validity and safety of placebo controls in randomized placebo-controlled surgical trials.

Central, MEDLINE, and EMBASE were systematically searched to identify randomized controlled trials comparing a surgical procedure to a placebo. “Surgical procedure” was defined as a medical procedure involving an incision with instruments. Placebo was defined as a blinded sham operation involving no change to the structural anatomy and without an expectable physiological response in the target body compartment.

Ten randomized placebo-controlled controlled surgical trials were included, all of them published in high-ranking medical journals (mean impact factor: 20.1). Eight of 10 failed to show statistical superiority of the experimental intervention. Serious adverse events did not differ between the groups (rate ratio [RR] 1.38, 95% confidence interval [CI]: 0.92–2.06, *P* = 0.46). None of the trials had a high risk of bias in any domain. The ethical justification for the use of a placebo control remained unclear in 2 trials.

Placebo-controlled surgical trials are feasible and provide high-quality data on efficacy of surgical treatments. The surgical placebo entails a considerable risk for study participants. Consequently, a placebo should be used only if justified by the clinical question and by methodological necessity. Based on the current evidence, a pragmatic proposal for the use of placebo controls in future randomized controlled surgical trials is made.

## INTRODUCTION

Randomized controlled, double-blind trials are considered to represent the state of the art in clinical research because this kind of study design minimizes bias and therefore limits the risk of invalid conclusions.^1^ Drug development follows a systematic testing process, passing through a number of highly regulated phases before eventual market approval.^2^ On the contrary, most surgical procedures have been introduced without reliable evaluation of their efficacy and safety, as there are no laws requiring testing of surgical strategies. Also, regulations for market approval of medical devices differ around the world.^3^ However, a step-up approach, known as the IDEAL recommendations,^4^ has been developed for use in surgical trials but has not yet been implemented broadly.

**FIGURE 1:**
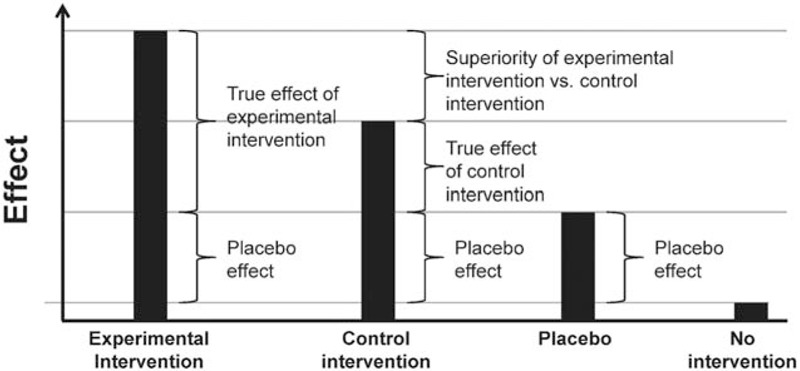
Visualization of conceptual effects of blinded interventions relative to no intervention.

In contrast to drug development, the placebo concept is not broadly implemented in surgical research. Basically, 3 different choices for a control group exist: no treatment, active agent, or a placebo control. Comparison against no treatment allows conclusions about efficacy of the experimental intervention compared with the natural history of a disease. Active control groups yield evidence on comparative efficacy.^5^ Finally, a placebo control group enables investigators to distinguish the true effect of an experimental intervention from a placebo effect (Figure 1).^6^ The placebo effect itself consists of several potential factors, resulting in a treatment effect despite the absence of a known active agent.^7^ The use of placebo in surgical trials involves several ethical and methodological challenges, which are mirrored by ongoing concerns and debates in the literature.^8–11^ An operation without indication legally constitutes a grievous bodily harm. Sham surgery (a fake operation) entails the general risks of surgical intervention, such as adverse effects of anesthesia and wound infection, while providing no apparent benefit to the patient.

The objective of this systematic review was to identify all available randomized placebo-controlled surgical trials to evaluate whether a placebo intervention can serve as a safe comparator and yield valid results in surgical trials. Furthermore, the methodological quality of the trials was examined to assess adherence to ethical justifications and methodological premises for the use of placebo controls in surgery.

## METHODS

This systematic review and meta-analysis was performed according to the recommendations of the PRISMA statement (Available at: www.prisma-statement.org. Accessed on August 25, 2015). The protocol of this systematic review was peer-reviewed by the Federal Ministry of Education and Research (BMBF), Germany (01KG1116). As no individual patient data were involved in the conduct of this systematic review and meta-analysis, no ethical approval was necessary.

### Research Objectives

This review had 2 objectives:

1. To investigate whether placebo controls can serve as a valid and safe instrument of trials in surgery, and to determine their scientific value. Internal validity was measured qualitatively by critical appraisal. For investigation of safety, a considerable risk was assumed if the complications were not lower in the placebo than the intervention group.
2. To explore adherence to ethical justifications and methodological premises for the use of placebo controls in surgical trials.

The sample of interest, therefore, comprised all randomized controlled trials (RCTs) using a placebo control group in any surgical discipline.

### Systematic Literature Search

A systematic literature search was performed according to the recommendations of the Cochrane Collaboration.^12^ The following databases were surveyed: Cochrane Library, MEDLINE (via PubMed), and EMBASE. The search strategy was based on a vocabulary thesaurus (MeSH or Emtree) in combination with text words: ((((((“placebo surgery”[tiab] OR “placebo procedure”[tw] OR “placebo procedures”[tiab] OR “placebo operation” [tiab]OR “sham surgery”[tiab] OR “sham surgeries” [tiab] OR “sham procedure”[tiab] OR “sham procedures”[tiab] OR “sham trial” [tiab] OR “simulated procedure” [tiab])) OR ((sham [tiab] OR “no treatment” [tiab]) AND (surgery [tiab] OR surgeries [tiab] OR surgical [tiab] OR surgically [tiab]OR laparoscop^∗^ [tiab])))) NOT (animal OR animals OR rat OR rats or rodent OR rodents OR rabbit OR rabbits))) AND (randomized controlled trial [pt] OR random^∗^). Additionally, a hand search of relevant cited publications was performed. The search was not restricted with regard to time, period, or language. The last search was performed on December 31st, 2014.

### Study Selection

RCTs from any surgical discipline were eligible if one of the study arms was a placebo control group. Surgery was defined as “a medical procedure involving an incision with instruments; performed to repair damage or arrest disease in a living body”.^13^ Trials evaluating endoscopic procedures, trials using a natural orifice for access, and interventional trials in cardiology or radiology using puncture as means of access were excluded.

In the absence of a convincing consensus definition of placebo^14^ in surgery, a conservative and strict definition was used. Placebo was defined by the 2 domains, that is, inertness and awareness. An intervention is said to be inert if there is no active agent that could change anatomy or induce a physiological response in a target body compartment, apart from the skin incision. Awareness of the perceived intervention arm is disabled by measurements of blinding.

Two reviewers (KG and PP) independently screened the abstract of every article identified by the search to determine its eligibility. The full texts of eligible articles were then assessed for definitive inclusion in qualitative and quantitative analysis. Any disagreement on eligibility was resolved with the aid of a third reviewer (MKD).

### Data Extraction

To answer the research questions, the following data were extracted: study characteristics; data on effect measure, with respect to the primary endpoint in the experimental intervention and placebo control group; and (serious) adverse events (SAE/AE), and their relation to the surgery performed.

Internal validity was critically appraised with the aid of the Cochrane Risk of Bias Tool.^12^ Moreover, success of blinding, that is, lack of awareness as a mandatory factor of the placebo effect, was assessed.

Ethical considerations were evaluated according to Horng and Miller.^15^ This assessment comprises 6 questions to estimate the risk-benefit ratio of the use of a placebo control in surgery. In addition, it classifies the use of placebo into 4 groups according to invasiveness (low, mild, intermediate, and significant).

### Statistical Methods

The extracted data on study characteristics, effect measures, safety, methodological quality, and ethical evaluation are presented descriptively. The primary endpoint of each included trial was identified irrespective of time of assessment or type of endpoint. In the case of >1 study arm with an intervention, each study arm was reported separately against the placebo group. Means and standard deviations for the intervention group and the placebo control group were extracted and reported in the case of a continuous primary endpoint. No distinction was made between final values and change scores. Absolute and relative numbers were identified for both treatment groups in the case of a binary primary endpoint. In addition, the *P* values resulting from the confirmatory analysis were extracted from the original publications and are reported.

An overall effect measure is not presented, as pooling was not justified because of the pronounced heterogeneity among the included trials with regard to indications, study population, and especially, clinical outcome measures. A forest plot is presented visualizing the effect measures as standardized mean difference and their 95% confidence intervals for all included trials. For reasons of simplicity, binary endpoints were converted to a standardized mean difference according to Chinn.^16^ Thus, all trial results could be visualized in a common forest plot, irrespective of the above-mentioned clinical heterogeneity. In addition, for the sake of consistent graphical presentation, the direction of treatment effect was changed if necessary, such that a negative effect measure consistently favored the intervention.

The numbers of SAE and AE are presented as rate ratios (RRs), that is, the rate of (S)AE in the intervention group in relation to the rate in the placebo group, with respect to the follow-up time in patient-months. Because of the use of a strict definition of placebo in this study, the placebo groups were homogenous and therefore the safety data were each pooled in a meta-analysis (contrary to efficacy data). A random-effect meta-analysis using the Mantel-Haenszel method was applied. An RR <1 indicates less (S)AE for the intervention group, whereas an RR >1 shows less (S)AE for the placebo control group.

The meta package in R (version 3.0.23.1.0, copyright © 2014, The R Foundation for Statistical Computing; meta package version 3.1–2) was used for all statistical analyses and forest plots.

## RESULTS

**FIGURE 2:**
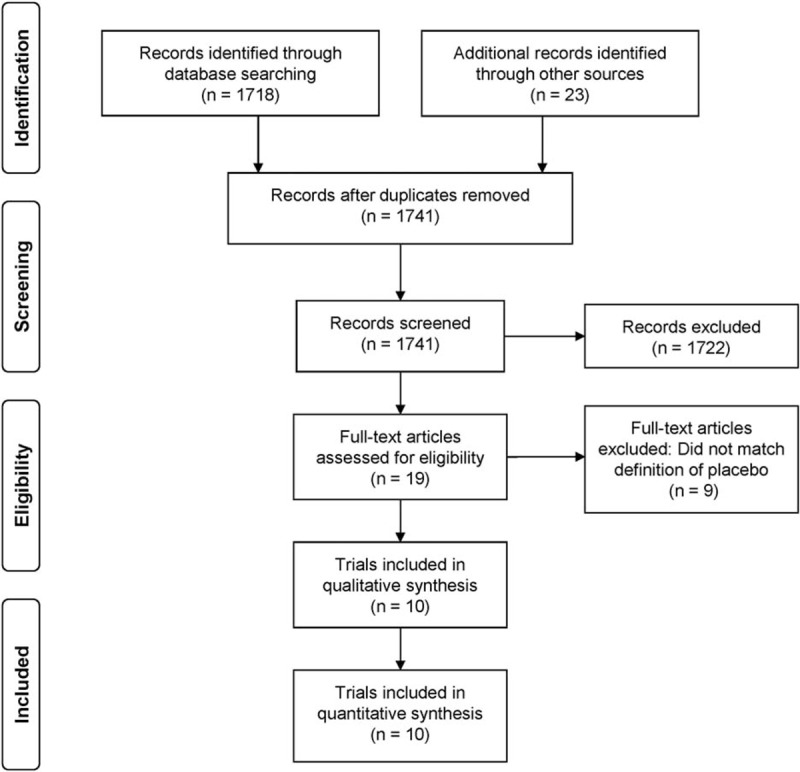
PRISMA flow diagram of included trials.

A total of 1741 articles were screened and the full text of 19 articles was analyzed. Of these, 9 articles were not eligible because they did not meet the criteria of a placebo as described above.^17–25^ Therefore, 10 eligible RCTs were included in a qualitative and quantitative analysis. A PRISMA flow diagram (Available at: www.prisma-statement.org. Accessed on August 25, 2015) is shown in Figure 2. All 10 of the trials included were published in English between 1996 and 2012. The publishing journals had a mean impact factor of 20.1 (range: 2.1–51.7) and were ranked in the top 25% of their respective surgical disciplines.

### Qualitative Analysis

Five of the RCTs belonged to the field of neurosurgery,^26–30^ 2 to orthopaedics,^31,32^ and 1 each to rheumatology,^33^ gynecology,^34^ and otorhinolaryngology.^35^

### Neurosurgery

Freed et al^26^ evaluated the possible benefit of transplantation of embryonic dopamine neurons into the putamen in patients experiencing typical symptoms of Parkinson disease (PD). In the control group, holes were drilled in the skull, but the dura was not penetrated. The primary endpoint was the change of disease severity according to a global rating score (GRS: −3.0 [worsening] to 3.0 [improvement]) after 12 months. The change in GRS revealed no significant difference between intervention and placebo group (0 ± 2.1 vs –0.4 ± 1.7, *P* = 0.62).

In the same way, Olanow et al^27^ evaluated bilateral mesencephalic fetal tissue transplantation into the putamen of PD patients, with tissue from either 4 fetuses or 1 fetus. The patients in the placebo group received partial burr holes not penetrating the inner table of the skull. Primary endpoint was the unified PD rating scale motor score (UPDRS 0–108; the higher the score, the more severe the disease), 2 years after intervention. No significant effect of transplantation compared with placebo was measured, although a slight effect was seen in the group that had transplantation from 4 fetuses (4.1 ± 4.8; −0.42 ± 2.8 vs 8.4 ± 5.5, *P* = 0.244).

Marks et al^28^ conducted a similar trial and delivered genes of AAV2-neurturin via bilateral transplantation into the putamen in PD patients. Patients in the placebo group received partial-thickness burr holes without penetration of the inner table of the skull. The primary endpoint was change in UPDRS (off medication) 12 months after intervention. No significant difference was found between the two groups (−7.21 ± 1.56 vs. −6.91 ± 2.12, *P* = 0.91).

Gross et al^29^ performed intrastriatal transplantation of human retinal pigment epithelial cells in patients with PD. Patients in the placebo group had partial-thickness burr holes. The primary endpoint was change in UPDRS motor score (off medication) at 12 months. Intervention and placebo group did not differ significantly (−10.5 ± 10.26 vs. −10.1 ± 12.26, *P* = 0.9).

Similar to Marks et al, LeWitt et al^30^ performed AAV2-glutamic acid decarboxylase gene therapy in patients with PD. The placebo was partial-thickness burr holes and the primary endpoint was UPDRS motor score (off medication) at 6 months. Significant improvement occurred in the intervention group (8.1 ± 1.7 vs 4.7 ± 1.5, *P* = 0.003).

### Orthopedic Surgery

Moseley et al^31,32^ performed 2 clinical trials with a placebo comparator, of which the first was a pilot trial. Two interventional groups (arthroscopic debridement and arthroscopic lavage) were compared with a placebo group (skin incision only) in patients with osteoarthritis of the knee. The primary endpoint in the confirmatory trial was pain, measured 24 months postoperatively on a knee-specific pain scale (KSPS 0–100; 0 = no pain, 100 = massive pain). Neither one of the interventional groups was superior to placebo (51.4 ± 23.2 and 53.7 ± 23.7 vs 51.6 ± 23.7; *P* = 0.64 and *P* = 0.96).

### Rheumatology

Davys et al^33^ investigated the potential benefit of debridement of plantar callosities on forefoot pain in patients with rheumatoid arthritis. In the placebo group, the debridement procedure was simulated with a blunt-edged scalpel. Forefoot pain was assessed using visual analogue scale (VAS). In both groups, VAS improved by 3 points (−3 ± 12.2 vs −3 ± 20.0, *P* = 0.48).

### Gynecological Surgery

In the RCT of Wei et al,^34^ the benefit of a midurethral sling to reduce incontinence after vaginal prolapse repair was investigated. The placebo group received skin incisions only. The primary endpoint was urinary incontinence at 12 months. The rate of incontinence was significantly lower in the treatment group than the placebo group (45/165 [27.3%] vs 74/172 [43.0%]; *P* < 0.001).

### Otorhinolaryngological Surgery

Koutsourelakis et al^35^ investigated the outcome of submucous resection of a deviated nasal septum in patients with obstructive sleep apnea syndrome. The placebo group received a simulated resection. The primary endpoint was the response rate after 3 months. There was no significant difference between the 2 groups (4/27 [14.8%] vs 0/22 [0%]; NS).

### Critical Appraisal

None of the 10 included RCTs had a high risk of bias in the domains of random sequence generation, allocation concealment, and incomplete outcome data. In 7 RCTs,^26,27,31–35^ no trial protocol was available and therefore the risk of bias in terms of selective reporting remains unclear. All trials blinded the patients to the respective intervention. One trial^33^ had a high risk of bias in the domain blinding. Patients were blinded to the intervention during the same-day assessment, but unblinded for the second assessment at 4 weeks, representing a possible reason for the lack of effect.

Only 3 of the 10 trials verified whether blinding of patients was successful. Moseley et al^31,32^ and LeWitt et al^30^ asked the patients which treatment they believed they had received. In these trials, patients were not able to correctly identify their treatment group.

### Ethical Considerations

According to the classification of Horng and Miller,^15^ the 5 neurosurgical trials entailed a significant level of risk because of their invasiveness into the skull. The 2 orthopedic trials and the gynecologic trial had an intermediate level of risk, whereas the otorhinolaryngological trial and the rheumatological trial involved mild and low risk, respectively.

According to Horng and Miller's “Ethical framework for the use of sham procedures in clinical trials” (Table 1), none of the trials investigated an irrelevant clinical question. Three of 10 trials^31,32,35^ explained explicitly the procedure of how patients were informed and gave their consent. In the other 7 trials, it can only be assumed that patients were informed of the misleading involved in the administration of a placebo control. For 2 trials,^26,27^ the data reported were insufficient for calculation of a sample size or any other estimation of a treatment effect. Nevertheless, a placebo control was used. In these 2 trials, the ethical justification for the use of a placebo control remained unclear. Among all included trials, only Moseley et al^31,32^ discussed the ethical aspects of the use of a placebo group in surgical trials.

**TABLE 1:**
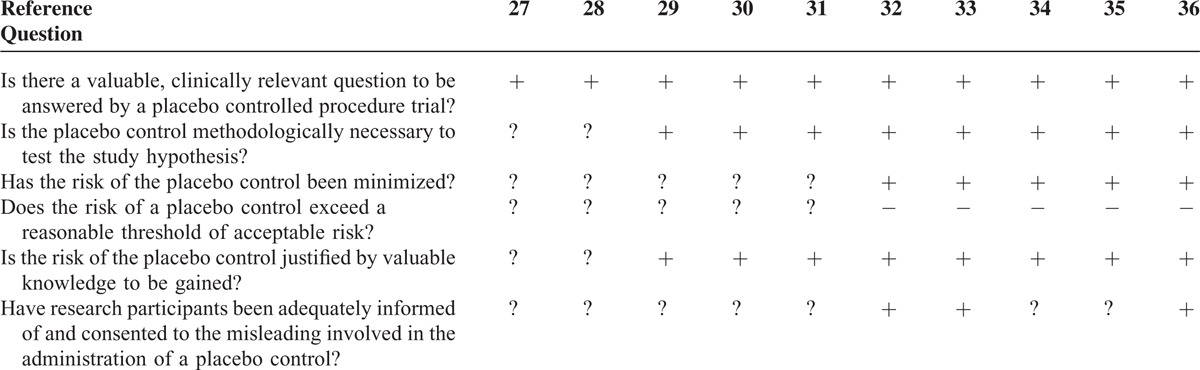
Ethical Framework for the Use of Placebo in Clinical Trials^15^

### Quantitative Analysis

The 10 RCTs included were all designed with a superiority hypothesis. A total of 840 patients (range: 10–337 patients per trial) were included, of whom 459 patients were allocated to the respective experimental intervention and 381 to the placebo group.

**FIGURE 3:**
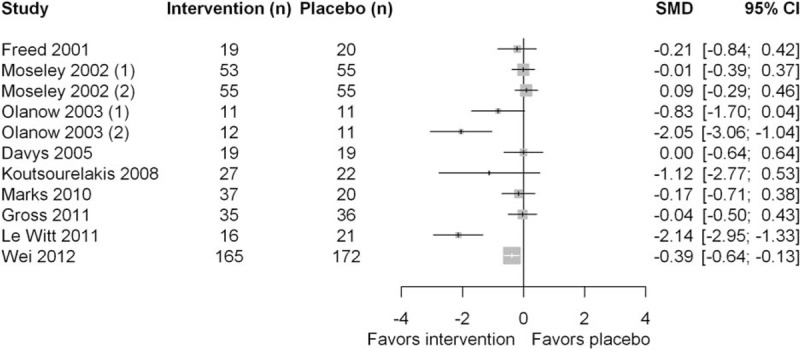
Forest plot comparing efficacy of primary endpoints.

Figure 3 displays a forest plot of primary endpoints. Two placebo-controlled RCTs^29,34^ and one subgroup analysis^27^ showed statistical superiority of the experimental intervention. A subgroup of 1 trial showed less effect of experimental intervention than placebo, although the difference was not significant.^32^ Quantification of the surgical placebo effect was not possible because none of the trials included a “no-treatment” group.

## SAFETY

**FIGURE 4:**
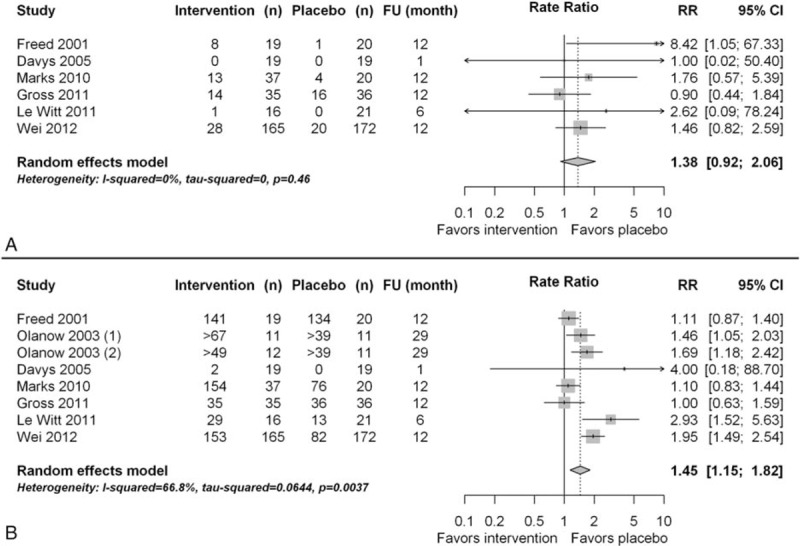
(A) Forest plot of serious adverse events; (B) Forest plot of adverse events.

Six of the 10 trials reported SAE.^26,28–30,33,34^ In the experimental intervention group, 64 of 291 patients experienced an SAE (0.22 SAE/patient), whereas in the placebo group, 41 of 288 patients had an SAE (0.14 SAE/patient). There was no statistically significant difference between the experimental intervention and the placebo group (RR 1.38, 95% confidence interval [CI]: 0.92–2.06, *P* = 0.46; Figure 4A). Only 1 SAE was reported to be related to the surgical intervention in the placebo group,^29^ compared with 13 surgery-related SAEs in the intervention group.^26,28,29^ However, 2 of the trials^33,34^ did not specify whether or not SAE was related to the surgical intervention.

Seven trials reported AE.^26–30,33,34^ In the experimental intervention group, 776 AEs occurred in 316 patients (2.5 AE/patient), and in the placebo group, 440 AEs occurred in 299 patients (1.5 AE/patient), representing a statistically significantly higher rate of AE in the experimental intervention group than in the placebo group (RR 1.45, 95% CI: 1.15–1.82, *P* = 0.004; Figure 4B). Only 2 trials reported the relation of AE to surgery.^29,30^ In these trials, 40 AEs were related to the surgical intervention in the placebo group compared with 48 AEs in the intervention group.

## DISCUSSION

This systematic review identified and analyzed all available randomized surgical trials with a placebo control group. For this purpose, a strict definition of placebo and surgery was used to achieve a representative sample. The objective was to draw evidence-based conclusions on placebo controls as a possible valid and safe comparator in surgical RCT.

The included RCT examined different surgical indications, interventions, and primary endpoints over observation periods of varying lengths. Therefore, a quantitative conclusion on the efficacy of surgical interventions compared with placebo surgery was neither justified nor feasible. The placebo effect of sham operations could not be quantified, as none of the included RCTs contained an additional “no treatment” study arm (Figure 1).

All trials included were based on data suggesting superiority of the respective experimental intervention. However, 8 of 10 trials showed no statistical superiority of the experimental intervention to the placebo control.^26–28,30–33,35^ Within these indications, the conduct of a placebo-controlled trial challenged their clinical implementation. None of the included trials reported problems related to the use of a placebo control group. Moreover, critical appraisal suggested a high internal validity of all trials investigated. Therefore, if indicated and properly conducted, randomized placebo-controlled trials are feasible and provide valid data on efficacy of surgical treatments.

Surgery-related risks were minimized in the interests of patient safety in each of the examined trials. However, the RR of SAE did not differ between the experimental intervention and the placebo group, that is, placebo controls were intended to be without effect; they caused no less serious harm to patients. This represents a significant contrast to placebo in drug development, wherein the potential harm in the placebo group is the lack of treatment. Therefore, the sham operation entails a considerable risk and should be used as a comparator only when justified by the clinical question^15^ and in presence of methodological necessity as discussed below.

Neither in surgery nor in another medical discipline a consensus definition of placebo exists. In addition, the placebo concept is controversially debated.^6,7,14^ Certainly, the definition of placebo used in this systematic review is strict and conservative, especially regarding inertness. Here, inertness means that apart from a skin incision, the sham operation should entail no changes of anatomy, as potential induction of a physiological response by penetration of the target body compartment has to be avoided. Hence, interventions such as laparoscopy and knee lavage may not be considered as inert and should more accurately be referred to as active control interventions with an uncertain effect. A strict definition would help to distinguish interventions with a potential placebo effect from active control interventions that may provoke physiological responses in the target body compartment, thus seriously confounding measurements of different effects (Figure 1).

Given the clear underrepresentation of surgical placebo-controlled trials in scientific literature, this systematic review implies that indications for placebo controls in surgery are rare. The existence of a publication bias in presence of the high number of published negative results seems very unlikely because restraining of positive results is very uncommon. Because of the invasive nature of surgery, the placebo concept is not readily transferable from drug development to surgical research, and the choice of control groups differs from the scenario of drug development.^36^ The absence of a clear definition of the surgical placebo and the lack of guidelines for the use of placebo controls in surgical RCT represents a huge limitation to surgical research.^37^

In the presence of another systematic review on the topic of use of placebo controls in the evaluation of surgery, it is important to delimit the present study from the existing one. The study by Wartolowska et al^11^ also included trials with endoscopic or radiologic interventions, resulting in a heterogeneous sample that most surgeons would not consider to be of their business. Moreover, important issues of the placebo concept, like blinding or inertness, were not addressed. Therefore, trials that claim any surgical control as “placebo” were uncritically included.^17,21,23–25^ For example, interventions like mastoidectomy or delayed surgery cannot be considered placebo controls in any way. Other trials that should have been included according to their selection criteria were not.^18–20^ Moreover, the occurrence of (S)AE was not meta-analyzed. Thus, the conclusion that placebo controls are much safer than the active treatment is ambiguous, and perhaps because of the inclusion of many low-risk interventions, not considered to be “surgery” by the present systematic review. This represents the major difference to the actual study, which presents sound evidence for hazards of surgical placebo controls. Furthermore, the systematic review by Wartolowska et al^11^ called for well-designed placebo-controlled surgical trials to challenge existing surgical procedures, and this ambitious demand deserves support. However, the absence of superiority of surgical interventions to placebo in the included trials in both systematic reviews must not be misinterpreted as a lack of effectiveness of surgical interventions in general. In broad fields of existing surgical interventions, for example, cancer surgery placebo-controls would be unethical and any change to clinical practice could be assumed. At this point the work by Wartolowska et al^11^ remains highly theoretical, whereas the present study includes pragmatic directions.

**FIGURE 5:**
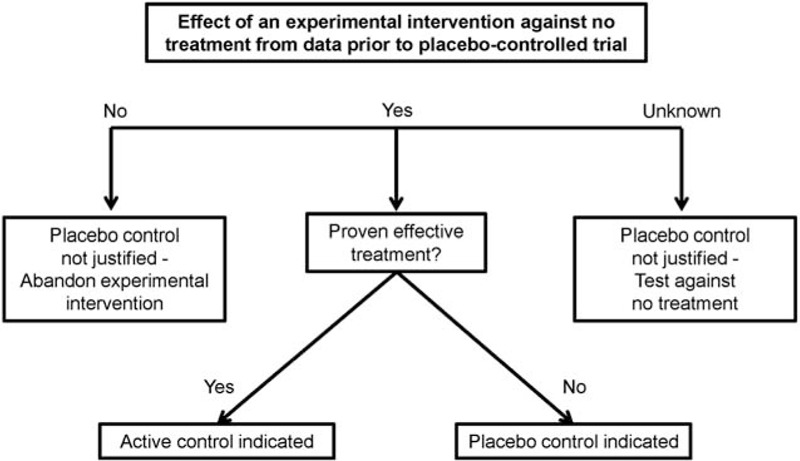
Decision diagram on justification of a placebo control in surgical trials depending on the data on an experimental intervention before randomized controlled trial.

Based on the presented evidence, this systematic review makes a pragmatic proposal for the use of placebo controls in surgical RCT (Table 2). As for any other clinical study, sound methodological and ethical premises are mandatory when conducting placebo-controlled surgical trials. The risk-benefit ratio must be balanced, and patients must give their informed consent for participation in the trial, including full information on the risks of the placebo intervention.^5^ Further, true clinical equipoise has to be assured, and in the existence of an effective treatment, an active rather than a placebo control group must be chosen.^38^ Preferably, the innovation under investigation should be at step 3 (assessment) according to the IDEAL-Recommendation.^4^ Moreover, data on the effect of an experimental intervention before an RCT are essential to be able to answer the questions posed in the “Ethical framework for the use of placebo in clinical trials.”^15^ In the event of known lack of effect of the experimental intervention in comparison with no treatment, a placebo control is not justified and the intervention under investigation should be abandoned. The optimal indication would be a known treatment effect versus no treatment, from data before the RCT and a lacking effective active comparator (Figure 5). As discussed above, the control group should not be uncritically referred to as a placebo group. Ensuring some kind of inertness in a surgical procedure represents a major challenge. In any case, the methods used in the placebo group have to be presented extensively, especially regarding blinding and inertness. Preferably, the success of measures taken to achieve blinding should be assessed. Although it is important to keep the risk of the placebo intervention as low as possible, unblinding of the patient must not occur because of minimization.

**TABLE 2:**
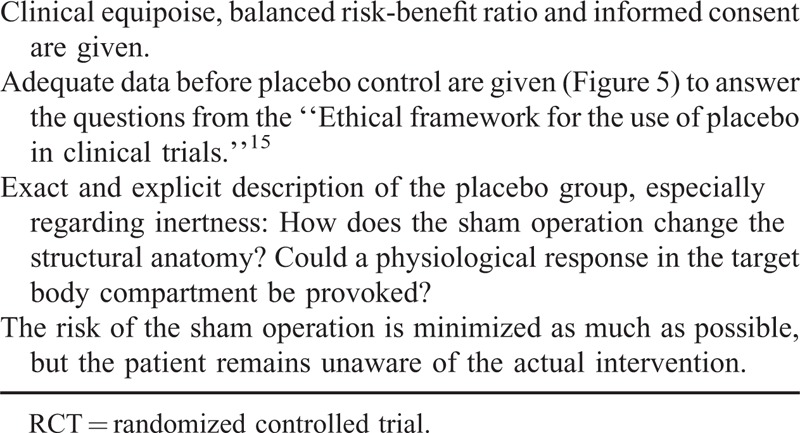
Summary of the Pragmatic Proposal for the Use of Placebo Controls in Surgical RCT

In summary, this review shows that properly conducted placebo-controlled trials can be feasible and are valid methodological instruments for the evaluation of efficacy of surgical interventions. Placebo controlled trials have a scientific value, and are mostly published in high-impact journals. However, surgical placebos entail a considerable risk for study participants and should be used only if justified by the clinical question and methodological necessity. The presented proposal should prove beneficial when implementing placebo controls in future surgical randomized controlled trials.
